# Physically sound, self-learning digital twins for sloshing fluids

**DOI:** 10.1371/journal.pone.0234569

**Published:** 2020-06-16

**Authors:** Beatriz Moya, Iciar Alfaro, David Gonzalez, Francisco Chinesta, Elías Cueto

**Affiliations:** 1 Aragon Institute of Engineering Research (I3A), Universidad de Zaragoza, Zaragoza, Spain; 2 ESI Chair and PIMM Lab, ENSAM ParisTech, Paris, France; Newcastle University, UNITED KINGDOM

## Abstract

In this paper, a novel self-learning digital twin strategy is developed for fluid sloshing phenomena. This class of problems is of utmost importance for robotic manipulation of fluids, for instance, or, in general, in simulation-assisted decision making. The proposed method infers the (linear or non-linear) constitutive behavior of the fluid from video sequences of the sloshing phenomena. Real-time prediction of the fluid response is obtained from a reduced order model (ROM) constructed by means of thermodynamics-informed data-driven learning. From these data, we aim to predict the future response of a twin fluid reacting to the movement of the real container. The constructed system is able to perform accurate forecasts of its future reactions to the movements of the containers. The system is completed with augmented reality techniques, so as to enable comparisons among the predicted result with the actual response of the same liquid and to provide the user with insightful information about the physics taking place.

## Introduction

Digital twins are tools to fully address real-world phenomena and seamlessly connect reality with virtual predictions. The abundance of data inputs allows to relate physical objects or processes with the simulation to obtain online meaningful results. The digital twin of the object of interest offers, in the end, a cost-effective real-time solution for taking decisions analogous to those that we would take interacting with the real process or object.

These virtual replicas have already shown their usefulness through their implementation across many kinds of industries and branches of science. In the field of robotics, they are the means for scene understanding and interaction to connect robots with the actual reality [1] [2].

In this paper, we focus on the development of a self-learning digital twin to predict the sloshing phenomenon of a fluid so as to equip an online decision-making system. By self-learning digital twin we mean a system that, rather than performing data assimilation (to determine the viscosity of the fluid, for instance) it is able to construct a physically correct, data-driven replica of a previously unseen fluid, regardless of its constitutive behavior.

We employ computer vision techniques to obtain data of the movement of the glasses where the real fluid and the replica are contained. Next, we feed the simulator engine with that information. Finally, the output is represented within a real container, that may be the one containing the fluid or not. We let the simulation run in the background so as to interpret the physics taking place.

Machine learning is a powerful tool to learn the behavior and underlying dynamics of a liquid [3]. Notwithstanding their effectiveness, physical compliance is still a central issue. In [4], the authors proposed an alternative data-driven approach to model the physics behind the sloshing dynamics as a regression guided by thermodynamic criteria.

Here, we substantially extend the algorithm so as to be able to construct replicas of a wide range of fluids for building a self-learning digital twin. We aim to prove that our method is also able to learn patterns of materials with non-linear behavior, with synthetic or real information from the scene.

## Related work

Fluid dynamics understanding based on machine learning is a rapidly growing field. It provides a deep insight into the physical complexity of flows and behaviors for their control and manipulation. Yunzhu Li et al. [3] study fluid simulation learnt from particle interaction. They developed the so-called dynamic particle interaction networks (DPI-Nets) in order to learn hierarchical interaction of the particles in which the objects of study have been discretized. They capture non linear behaviors in deformable and fluid bodies for control purposes. Schenck and Fox [2] also employed convolutional networks to learn the physics of pouring liquids. Noteworthy, these predictions have no guarantee to comply with the laws of Navier-Stokes, for instance. The same problem arises in the work by Kennedy et al. [5], where approximate particle methods are employed to simulate pouring. In general, in the field of robotics, existing approaches employ approximate methods to overcome the very stringent real-time constraints imposed by closed-loop fluid simulation. This need for very fast simulation results has prevented existing methods from accurately satisfying basic conservation laws such as Navier-Stokes of the laws of thermodynamics. The present work overcomes these limitations and guarantees the satisfaction of the laws of thermodynamics while avoiding the need of solving Navier-Stokes equations.

Very few references analyze the possibility of applying the concept of digital twin to fluid mechanics. Among them, we cite the application to drilling fluids in the field of geology, see [6], or an approach for the oil and gas industry [7].

Brunton et al. [8] make a review of these recent advances in machine learning applied to fluid mechanics and dynamics. It stands out the need for these systems to overcome challenges such as *generalization* and *interpretation*. Particularly, there is a growing interest on machine learning strategies that satisfy well-known principles of physics and do not construct black-box models whose application to previously unseen data does not guarantee to comply with basic principles such as energy conservation or equilibrium.

In order to overcome these limitations, the machine learning community has recently taken a step forward physically-informed learning [9], whose application field ranges from neural networks [10] [11] [3] [12] to linear regression learning [13] [14]. This new perspective of machine learning aims to guide the learning process with criteria that fulfills the laws of physics. As a result, we achieve a higher degree of accuracy, as well as generality and interpretability. In turn, those models still entail sometimes a high computational cost difficult to surmount. Under this rationale, real-time constraints have not been—to the best of our knowledge—attained.

Real-time digital twins offer applications for continuously changing, monitored systems [15]. For this purpose, time constraints must be satisfied. Reduced order models supply digital twins with tools to face such disadvantage [16] [17] [18] [19]. Kapteyn et al. [20], for instance, work on physically-constrained digital twins developed in a reduced order space for aircraft replanning. The model for each topology is defined in a reduced basis, giving support for immediate decision making.

Twins continuously incorporate features to improve interpretation and application. Augmented reality (AR), for instance, enables the contextualization of the twin in the physical world. Consequently, interaction between reality and the replica, as well as user control and interpretation, are straightforward. AR tools have also been successfully implemented for non-rigid representation, such as aerodynamics [21], or deformable objects [16]. On the other and, existing digital twins for sloshing problems revealed limitations, such time computing and localization in the scene, that inhibit their implementation [22].

## Method

In this work we suggest a data-driven twin which performs a thermodynamically admissible emulation of sloshing dynamics. To this end, the method consists of the merge of three stages: data assimilation, interpretation and representation. The final goal is to predict the next state of a fluid, which could be unknown, from interpretable data of the scene. Some of the fluids employed to learn the dynamics show viscoelastic properties. In addition, the twin needs to operate at a frequency of 30 Hz or faster (the one at which standard cameras operate). The result is an AR digital twin convergent and synchronized with reality for optimal control and decision making.

### Integration from data without the need to solve Navier-Stokes

Machine learning abstracts insights about the physics from given data. A priori, they are unknown, but the algorithm finally learns the appropriate correlations to fit an accurate model.

Our digital twin is modeled as a time-evolution problem, expressed as a function of a set of variables $z_{t}=z\left(t\right)\in S\subset R^{D},$ with *D* representing the full dimensionality of the problem. These variables describe the energy and entropy evolution of the fluid. If we know them at discrete time steps, we will try to find patterns so as to predict future states of the liquid. With this integration scheme, given new real or synthetic measurements of an unseen trajectory, the sloshing dynamics will be accurately reconstructed in real time.

This approach employs the so-called General Equation for Non-Equilibrium Reversible-Irreversible Coupling, GENERIC [23]. This methodology has been employed satisfactorily in several works for learning models [24] [25] [4], as well as corrections to existing models [26], from data.

GENERIC arises as a general thermodynamic framework to describe mesoscopic dynamics. Actually, it can be seen as the formulation that describes the process of obtention of less detailed models, reduced from models involving more details (more degrees of freedom). The phase portrait of a reduced model is seen under this theory as a pattern in the phase portrait of the detailed model [27]. As a result, it expresses the dynamics in terms of the mesoscopic variables ***z***, that lead the evolution of energy and entropy of a system as:

$$\begin{array}{c} \frac{dz}{dt}=L\frac{\partial E}{\partial z}+M\frac{\partial S}{\partial z}, \end{array}$$ (1)

where ***L*** and ***M*** are the poisson and friction matrices, and ∇*E* and ∇*S* the gradients of energy and entropy, respectively. We distinguish two main parts in this expression, one related to the conservative part of the dynamics (***L***∇*E*), and another representing the posible dissipation of the system (***M***∇*S*).

We also ensure thermodynamic consistency by imposing the so-called degeneracy conditions:

$$\begin{array}{c} L\frac{\partial S}{\partial z}=0,\;M\frac{\partial E}{\partial z}=0, \end{array}$$ (2)

that establish that the energy functional does not contribute to dissipation mechanisms, while entropy does not contribute to reversible mechanics [28].

We guarantee both impositions by forcing ***L*** to be the classical symplectic, skew symmetric, matrix of Hamiltonian mechanics, and ***M*** symmetric, positive semi definite. With the former restriction we ensure energy conservation:

$$\begin{array}{c} \dot{E}\left(z\right)=\nabla E\left(z\right)\cdot \dot{z}=\nabla E\left(z\right)\cdot L\left(z\right)\nabla E\left(z\right)+\nabla E\left(z\right)\cdot M\nabla S\left(z\right)=0, \end{array}$$ (3)

and with the latter, entropy generation

$$\begin{array}{c} \dot{S}\left(z\right)=\nabla S\left(z\right)\cdot \dot{z}=\nabla S\left(z\right)\cdot L\left(z\right)\nabla E\left(z\right)+\nabla S\left(z\right)\cdot M\nabla S\left(z\right)\geq 0. \end{array}$$ (4)

We assume that the sloshing dynamics of each fluid evolve on a finite-dimensional, smooth and real manifold $M\in R^{D}$, reconstructed from synthetic data. In this approach, the discretized, pseudo-experimental measurements of the state variables allow to build an also discretized expression of GENERIC:

$$\begin{array}{c} \frac{z_{n+1}-z_{n}}{\Delta t}=L\left(z_{n+1}\right)\text{DE}\left(z_{n+1}\right)+M\left(z_{n+1}\right)\text{DS}\left(z_{n+1}\right), \end{array}$$ (5)

from which we can also learn new trajectories. Here, L(***z***_*n*+1_), DE(***z***_*n*+1_), M(***z***_*n*+1_) and DS(***z***_*n*+1_) represent the discretized approaches to their original counterparts. Note also that ∇*E* and ∇*S* can be approximated, in the finite element style, by piece-wise polynomials so that:

$$\begin{array}{c} \nabla E=Az,\nabla S=Bz, \end{array}$$

with ***A*** and ***B*** two matrices of shape function derivatives. This makes ***L***, ***M***, ***A*** and ***B*** the objective of the following (piece-wise) linear regression procedure:

$$\begin{array}{c} \mu^{*}=\left\{L,M,A,B\right\}=\underset{\mu }{\text{arg}\;\text{min}}∥z\left(\mu \right)-z^{\text{meas}}∥. \end{array}$$ (6)

**Fig 1:**
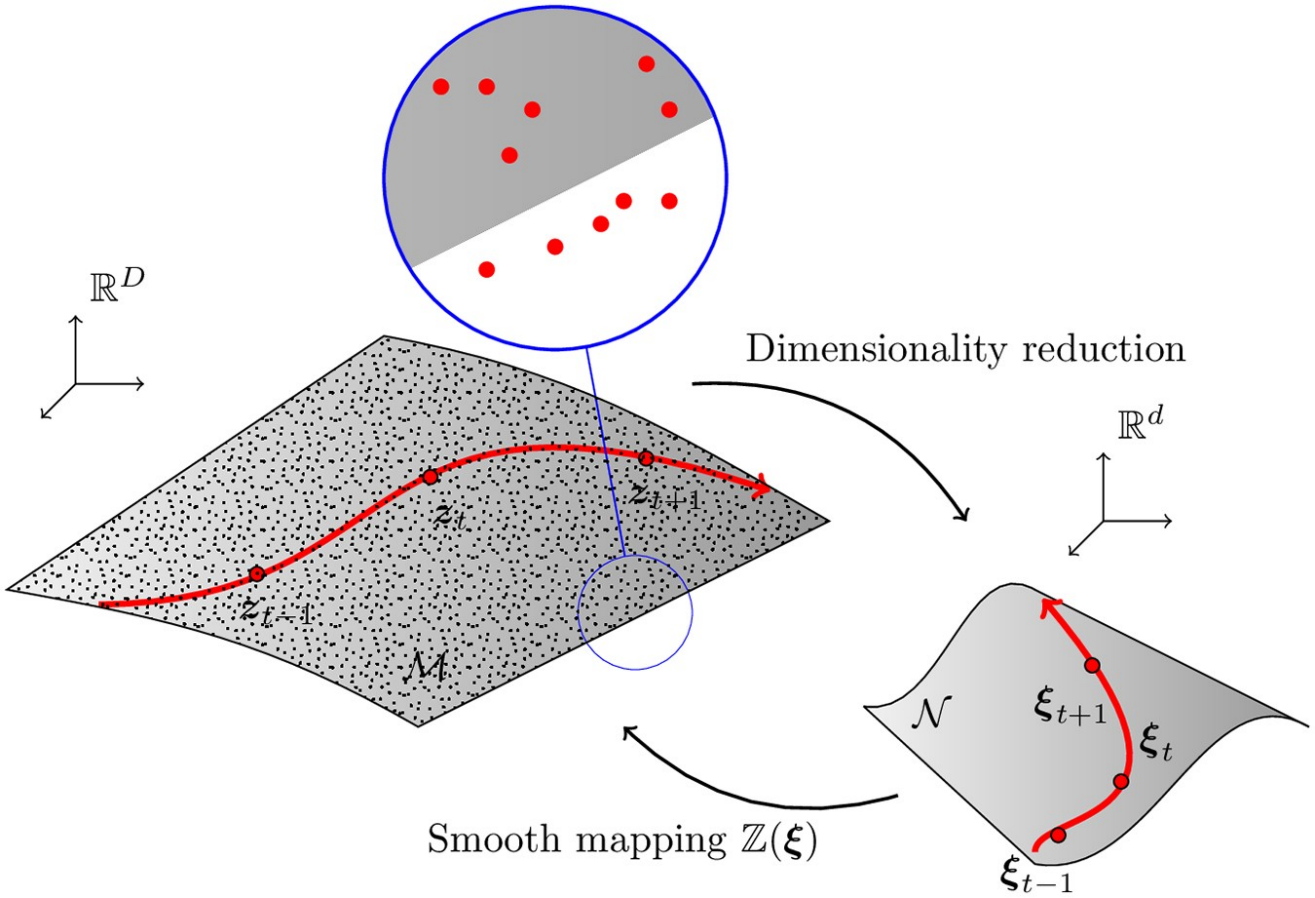
We assume the existence of a slow manifold M on which each fluid lives. We extract synthetic data as vectors in $R^{D}$ from computational simulations. These data are possibly noisy, see the zoomed detail. An example sloshing phenomenon is represented as the red line in phase space. Given the inherent high-dimensionality of the manifold, nonlinear dimensionality reduction techniques will also be applied. This will project the data to a lower dimensionality embedding space in $R^{d}$, with *d* ≪ *D*. We develop structure-preserving integration schemes to integrate the evolution of the system in this low-order manifold. These are then mapped back to the physical space in $R^{D}$ so as to obtain meaningful results.

Nonetheless, the optimization and calculations are still computationally demanding. In order to achieve real time performance for our digital twin, we make use of model order reduction techniques to find a reduced order manifold $N\in R^{d}$, where *d* ≪ *D*. Fig 1 sketches our approach. On this reduced manifold, we will preserve the important features of the dynamics expressed in a new system of latent variables.

In [4], the authors showed the efectiveness of non-linear model order reduction techniques (namely, LLE [29] and TDA [30]) for this problem. Following these results, we have employed *k*-PCA (kernel Principal Component Analysis) [31] to distill the reduced order manifold of each fluid.

In our problem, we have a data matrix ***Z***. Each column ***z***_*i*_, *i* = 1, …, *n* is a snapshot, a vector of state variables that represent the state of the fluid at a specific time instant. For each fluid, we have a total of *n* snapshots.

$$\begin{array}{c} {}[\begin{array}{cccc} | & | & & |\\ z_{1} & z_{2} & \cdots & z_{n}\\ | & | & & | \end{array}]=Z\in R^{D\times n}. \end{array}$$

Given a matrix ***Z*** snapshots, we compute the product ***S*** = ***Z***^*T*^ ***Z*** to obtain the matrix of pairwise scalar products. The key hypothesis of *k*-PCA is that the projection of the points to a new space $\phi :M\subset R^{D}\to R^{Q}$, where *Q* is the new dimension, probably higher than the current space, can result to be linearly separable. We apply PCA in $R^{Q}$. As a result, we obtain the most relevant nonlinear principal components of ***Z*** and thus a projection to a much lower dimensional manifold. With this method we successfully embedded then into a three dimensional system $N\subseteq R^{d=3}$.

The discretized GENERIC formulation is also consistent in the low dimensional manifold we have built [32]. L and M are squared matrices whose shape we frequently know from the description of the problem we model—there is a vast literature in the field [33] [34] [25] [28] [23]—. Nevertheless, they cannot be projected to the non-linear low dimensional manifold where the database has been projected, where we risk to loss the rich thermodynamic structure induced by Eqs (3) and (4). As a result, they are also the objective of the regression procedure in the reduced manifold.

### Experimental and pseudo-experimental data

GENERIC learns the sloshing dynamics of the liquids from the measurements of the state variables at discrete time steps. We have obtained pseudo-experimental data from simulations performed by employing Smoothed Particle Hydrodynamics (SPH, [35]) for training and test. Once it reaches the test phase, the twin learns previously unseen trajectories from experimental data.

**Fig 2:**
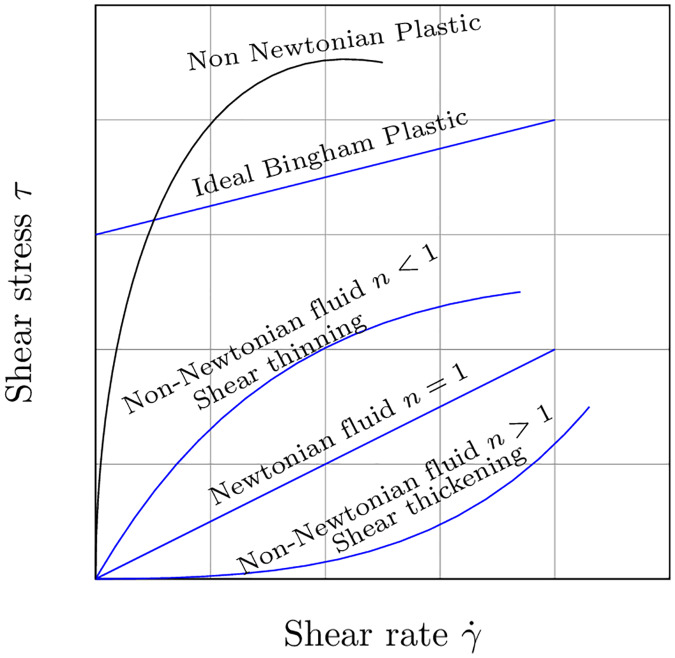
Standard classification of the fluid families considered herein. In the case of Newtonian fluids, their properties are constant over time and show a linear response. Their flow index *n* is then set to 1, and their yield stress to *τ*_0_ = 0. In contrast, shear thinning fluids start flowing when the stimulus is greater than the yield stress *τ*_0_ > 0. For these fluids, *n* > 1. In this work, fluids whose behavior can be assimilated to shear thinning have been considered. Fluids that incorporate some kind of plastic behavior need for a special treatment and have not been considered yet.

Here, we generalize the scope of our first work [4] to identify the dynamics of a wider database. It has been specifically constructed with pseudo-experimental results of water, glycerine, butter, honey, mayonnaise and chocolate. Our intention is to cover different viscosities and densities, as well as behaviors. The different fluid constitutive models are sketched in Fig 2. Chocolate, mayonnaise and blood are considered shear thinning [36] [37] [38] [39]. We have selected the Herschel-Bulkley model [40] to reproduce their rheology. It follows the next expression:

$$\begin{array}{c} \tau \left(t\right)=k{\dot{\gamma }}^{n}\left(t\right)+\tau_{0}, \end{array}$$

in which ***τ*** is the shear stress, *k* the consistency index, $\dot{\gamma }$ the shear rate, *n* the flow index, and ***τ***_0_ the yield stress.

We have simulated four different sloshing trajectories for each liquid assuming Smooth Particle Hydrodynamics discretization [35], according to the parameters in Table 1. This approach provides discrete data of the properties of each particle. Data have been extracted from snapshots taken every 0.005 seconds to capture the important features of the dynamics and avoid the effect of overfitting as well.

**Table 1:** Characteristics of the fluids considered in this work.

Fluids	*k* (Pa ⋅ s)	*n*	*τ*_0_ (Pa)
Glycerine	0.95	1	-
Blood	0.017	0.708	-
Mayonnaise	45.40	0.495	98.18
Melted chocolate	5.764	0.697	9.096

When learning Newtonian fluids, we only need the position ***r***_*j*_, velocity ***v***_*j*_ and internal energy *E*_*j*_ of each particle at discrete time instants. However, non-Newtonian fluids are not fully described with these state variables [41]. Some of the liquids we attempt to describe here have viscoelastic properties, and this behavior is captured by including the stress tensor ***τ***_*j*_—or a related magnitude—of each particle in the set of state variables. The fluid is then characterized at each time by a vector of these particle variables, such as

$$\begin{array}{c} S=\{z=\left(r_{j},v_{j},E_{j},\tau_{j},j=1,2,\ldots ,M\right)\in {\left(R^{3}\times R^{3}\times R\times R^{6}\right)}^{M}\}, \end{array}$$

for every *n* > 1 equally spaced time steps in the interval (0, *T*] of each simulation. The fluid volume has been discretized into *M* = 2134 particles. If we have 13 degrees of freedom per particle, the full dimensionality of each snapshot vector is *D* = 27742.

Once the integrator has been built, we learn an unseen trajectory from a new vector of state variables, which establishes the initial conditions to emulate the sloshing dynamics. When running the digital twin, this information comes from the scene.

We make use of computer vision techniques to track the movement of the glass. For these purpose, we have added texture to it to track its features. See the details in the Appendix. This information has to be converted to an interpretable input for the integrator. The simulations of the database have been obtained by defining different input velocities of the glass to trigger the sloshing. Therefore, we relate the initial state of the virtual fluid with the tracked velocity of the container as an interpolation. With this information, we can perform the computation of the next state of the liquid.

There are some features of the algorithm that also require data acquisition of the free surface. Albeit the use of sensors is widely spread, image analysis is an attractive option for experimental data acquisition. The use of RGB Depth cameras is common used for this purpose [42] [43]. However, due to its lack of texture, it is a difficult task that could need to be supported with CNN for accurate tracking [44].

**Fig 3:**
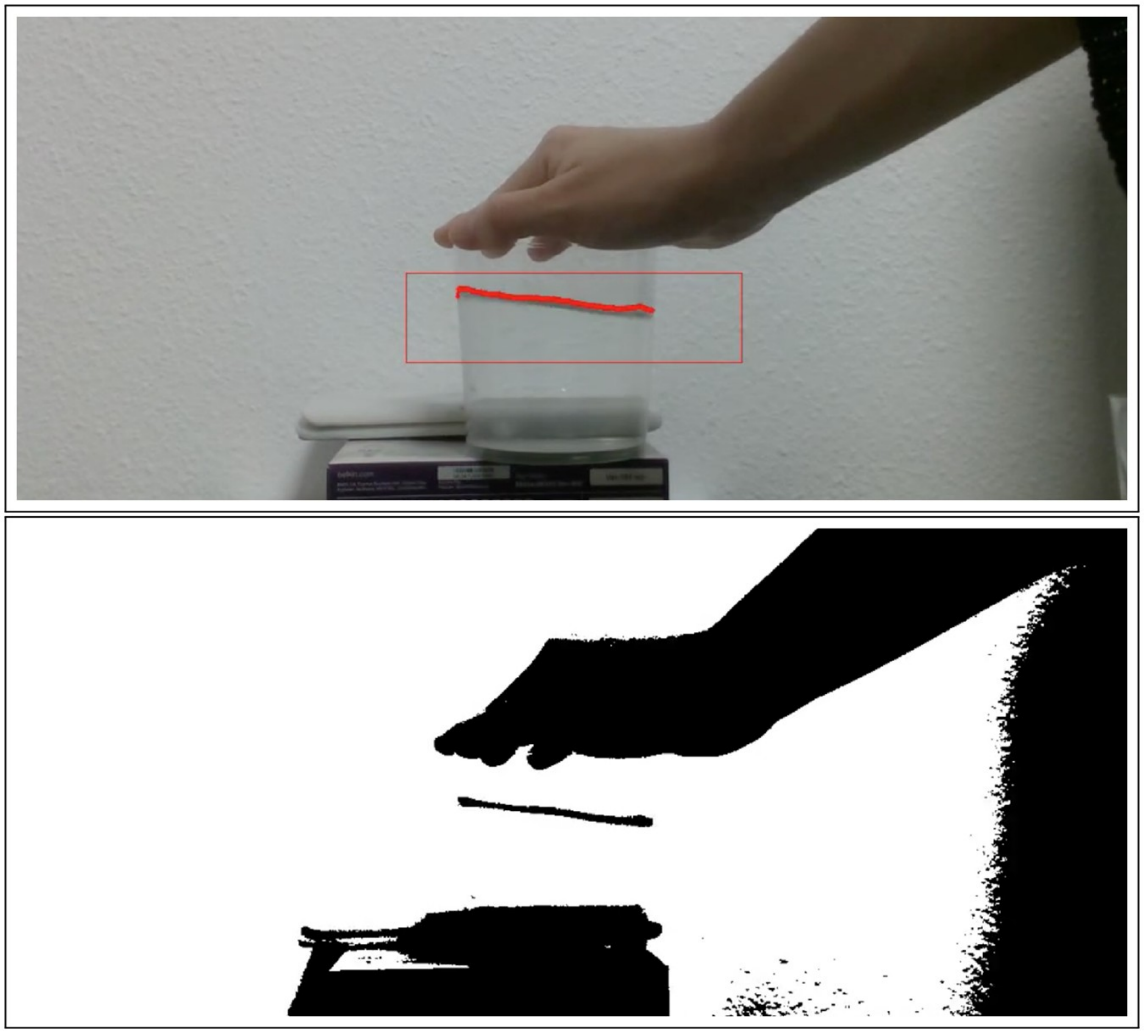
Example of frame binarization. Original frame (top) versus binarized one (bottom). The picture is transformed firstly to gray scale for gentle binarization. Noise is also filtered to detect a smooth surface, which is highlighted in red in the top figure.

**Fig 4:**
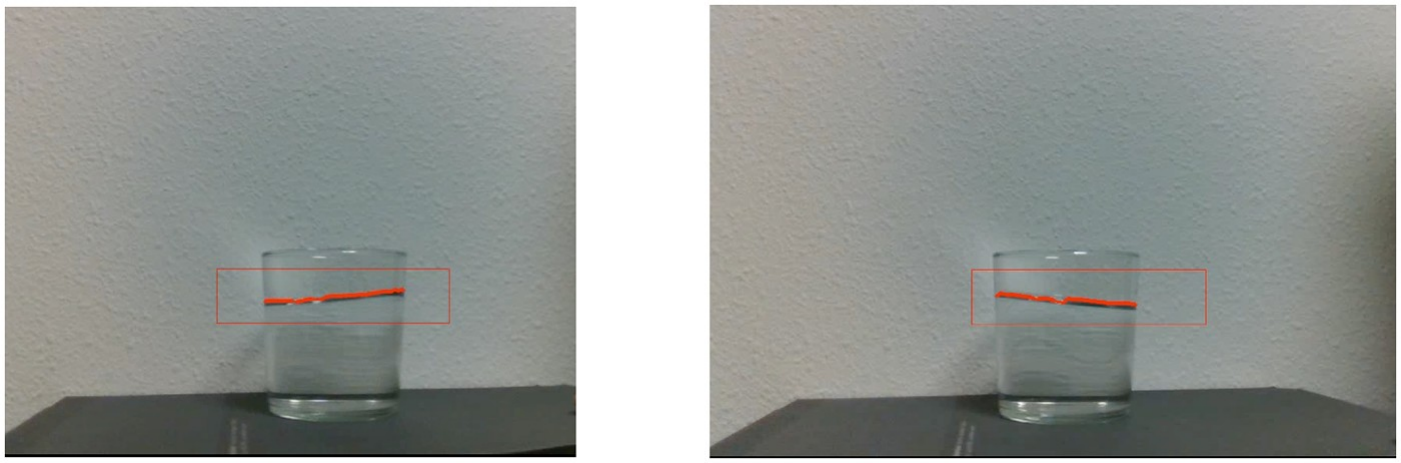
Free surface detection and tracking in video sequence. The points selected to belong to the free surface are highlighted in red over the original frame for verification.

We followed an approach similar to the one employed in [45]. We analyze the color gradient of the binarized frame (Fig 3). There is a noticeable change between the free surface and the background. Therefore, the points in the boundary where there is a color gradient are considered free surface. These points are stored, tracked, and augmented in the image for user interaction and verification (Fig 4).

### Fluid clustering

The digital twin should be able to mimic the behavior of any type of fluid, not necessarily in the database. Then, in future applications, it must perceive and recognize the fluid as well as explain what it is watching in understandable terms for a human. This is called *interpretable machine learning* [46].

We expect to classify the fluid with only a few observations and, knowing which fluid it is—or interpolating between the closest neighbors in the phase space—, make the calculations in the appropriate reduced order manifold.

The high dimensionality of the problem would make unfeasible to perform the classification as fast as the application requires. We address this problem by applying model order reduction techniques. This allows us to perform the classification in a lower-dimensional manifold. Dimensionality reduction is a common preprocessing step for classification tasks. This process entails a noticeable time reduction in the classification. In addition, if the dataset has a lower dimensional structure, we avoid the noise of the large dimensional set, what results in an improvement of the accuracy.

**Fig 5:**
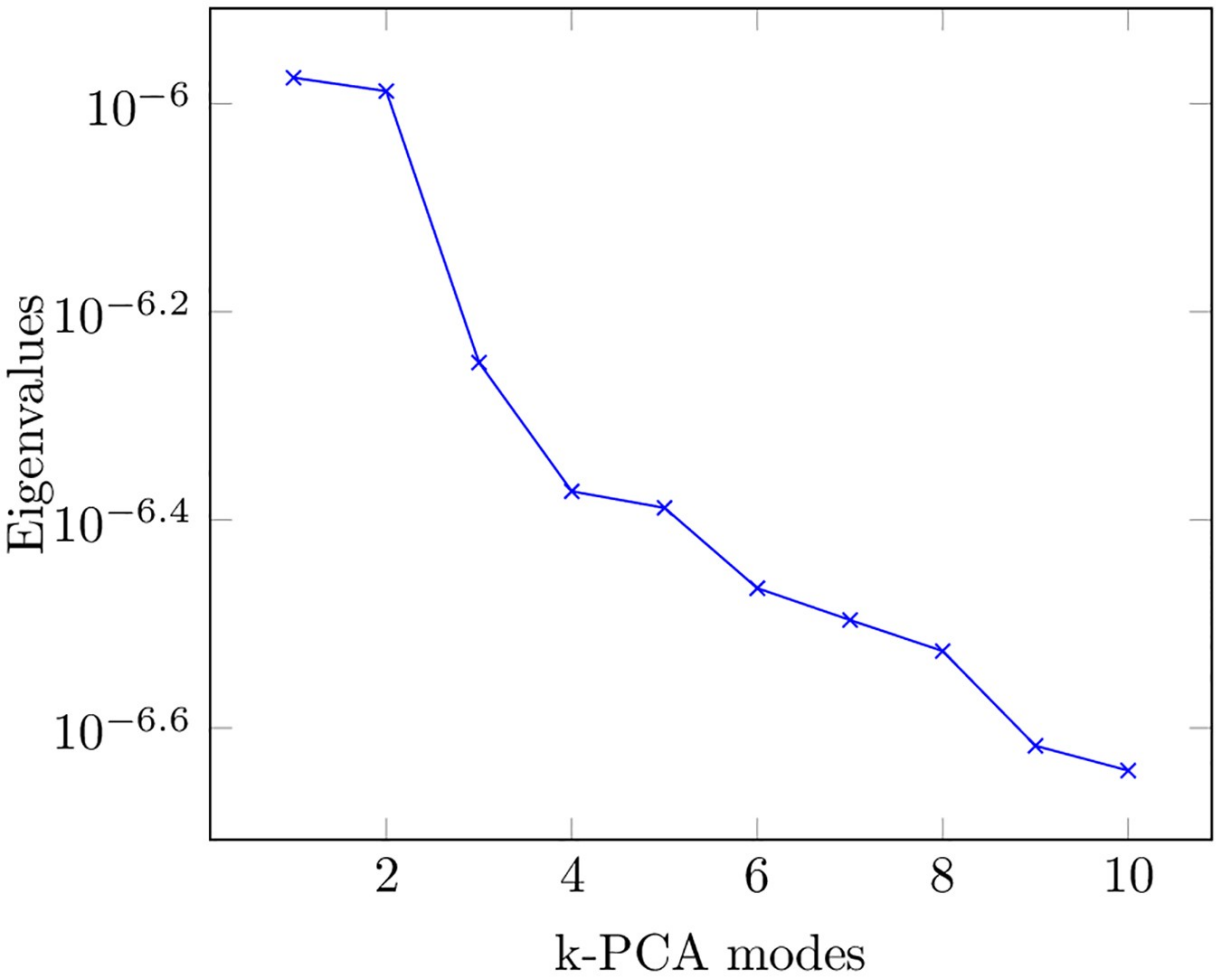
This picture represents the evolution of the eigenvalues for the first 10 *k*-PCA modes. We distinguish three modes that stand out with regard to the others. This fact justifies the reduction to the embedded manifold. As a result, we aim to provide with a more manageable and efficient system for classification.

**Fig 6:**
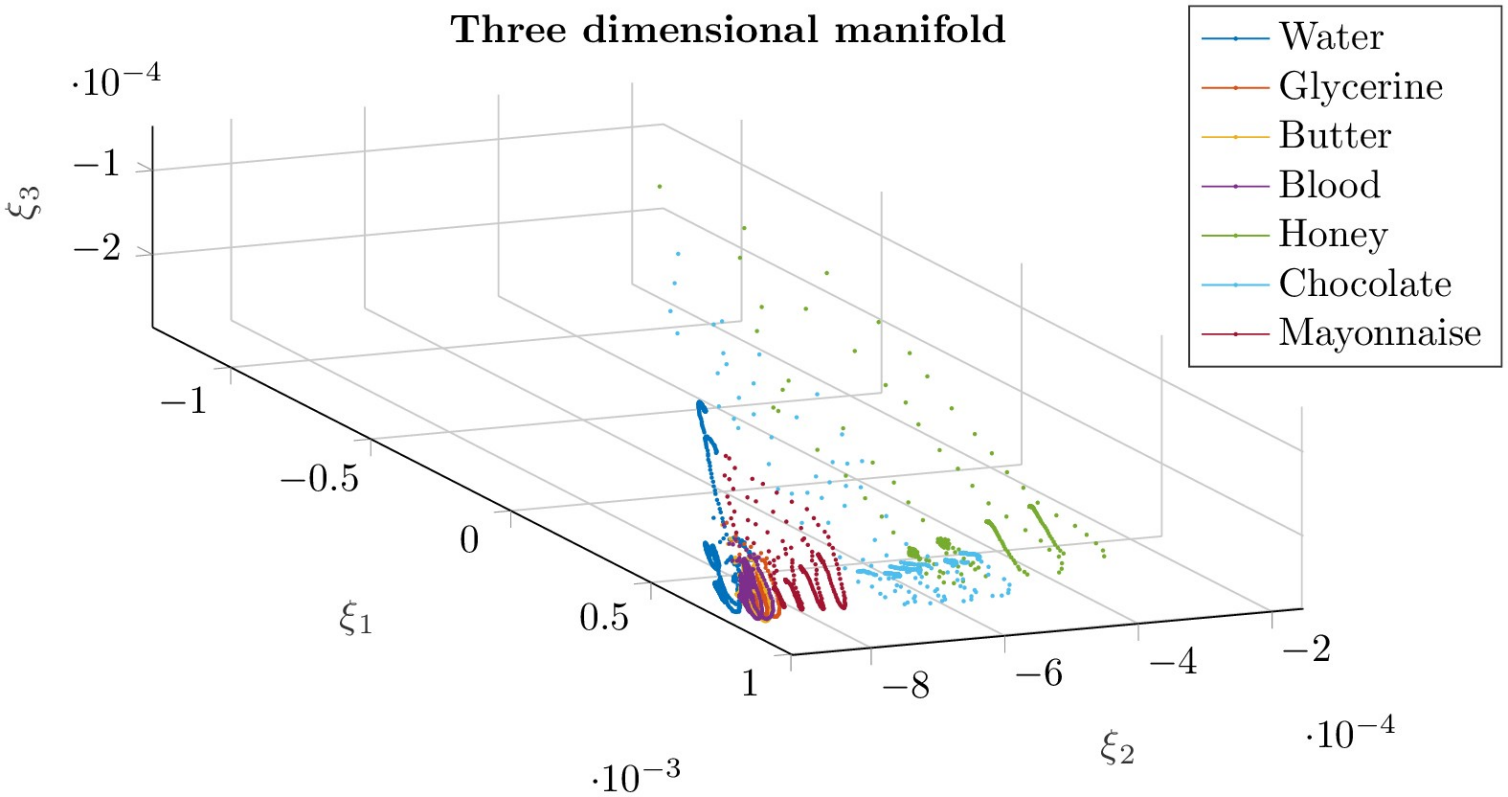
By employing *k*-PCA we reach a manifold of 3 dimensions where the different fluids, represented by one color each, remain clustered.

We choose again *k*-PCA to perform the reduction of our problem [31]. *k*-PCA enables us to project the data onto a low dimensional manifold. In our case, and despite the smooth decay in the eigenvalues, see Fig 5, some 3 dimensions have shown to provide with good clustering results. The different types of fluids remain clustered in the new projection, as can be seen in Fig 6. *k*-PCA is able to unveil the features that make the behavior of each fluid unique with respect to the others. We expect this fact to be advantageous for the classification process.

In our case, we employ random forests [47] for classifying our dataset. This technique consists of constructing several decision trees in different subspaces of the training set to generalize the classification and, as a result, avoid the overfitting that usually appears in single regression tree techniques.

We also apply *k*-fold cross validation as part of the learning process. The method suggests to split the database into two parts, one assigned for training and the rest saved for testing. This process is repeated iteratively *k* loops, changing the distribution data of training and test sets, to improve the fitting.

We need enough data to recognize the underlying trend, but we also need to leave sufficient for testing so as to avoid high variance error. According to this criteria, we establish a relation of 80% of snapshots for training, and 20% for test, in our algorithm. We trained the model following a cross validation scheme in *k* = 5 iterations.

Overall, the results obtained from the classification algorithm showed a good performance. High accuracy was achieved analyzing both global results as well as the error obtained for each fluid individually (Table 2). With this result, we consider the model valid for our problem, as well as for decision making applications.

**Table 2:** Results obtained from classification after random forest training with k-fold cross validation.

Individual Accuracy	Water	99.17%
	Glycerine	97.32%
	Butter	92.88%
	Blood	91.07%
	Honey	91.07%
	Mayonaise	91.07%
	Chocolate	91.07%
Global Accuracy	95.93%	
Precission	99.17%	
Recall	99.17%	

### Augmented reality

The prediction performed by the integration scheme is presented to the user through augmented reality (AR). It provides a user friendly interface from control and understanding. This technology is usually employed for rigid representation, i.e. the virtual object does not interact with any real stimulus. Our twin is deformable, and interacts actively with the scene.

We employ the tracking information previously obtained for precise placing of the augmented liquid. In consequence, it continuously updates and shows real time connection and interaction with the glass.

AR representation shows the position of the particles and the free surface. In addition, it can augment the representation of the liquid showing additional information, such as the velocity field.

## Results and discussion

We have tested the online performance of the twin to evaluate the implementation of the algorithm in conjunction with the computer vision techniques that we employ for data extraction. We obtained positive results in the merging of both elements. Trajectory generation and augmented representation is done online, and coupled with the video. As a result, we perform real time calculation and representation.

The replica of the liquid has been also compared with a glass filled with a liquid of the same type. Both containers are subjected to the same forces. Qualitatively, liquids are synchronized. Nevertheless, the movement of the digital twin seems to be a bit more amplified.

**Fig 7:**
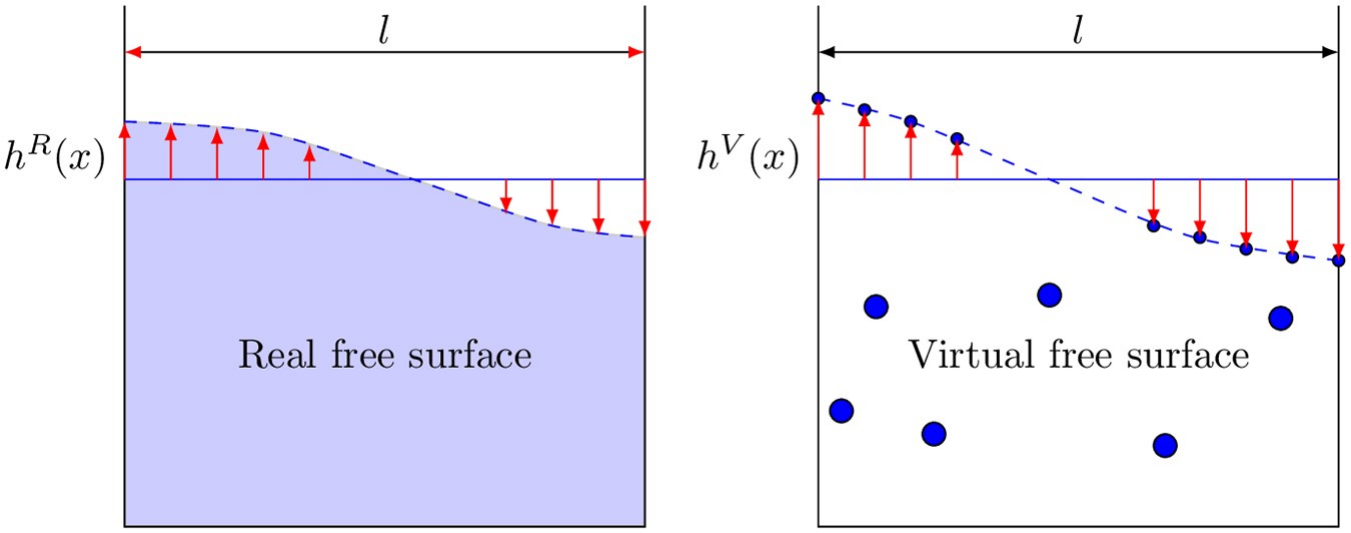
Representation of the quantifiable comparison of the real liquid and the replica. Free surface is defined as a function of its height at different points. These heights are compared in a same snapshot to evaluate the reconstruction error.

While previous approaches in the field (notably [44] [2] [5]) report qualitative performance measurements only, we have also tried to provide with quantifiable results to perform accurately the experimental validation. We quantify the error in the reproduction of the free surface reconstruction, defined as the integral of the differences between the heights regarding a medium line, see Fig 7:

$$\begin{array}{c} e=\frac{1}{l}\int_{l}(|h^{R}\left(x\right)-h^{V}\left(x\right)|)dx, \end{array}$$ (7)

with *R* and *V* representing the real (physical) height and the virtual one, respectively, see.

**Fig 8:**
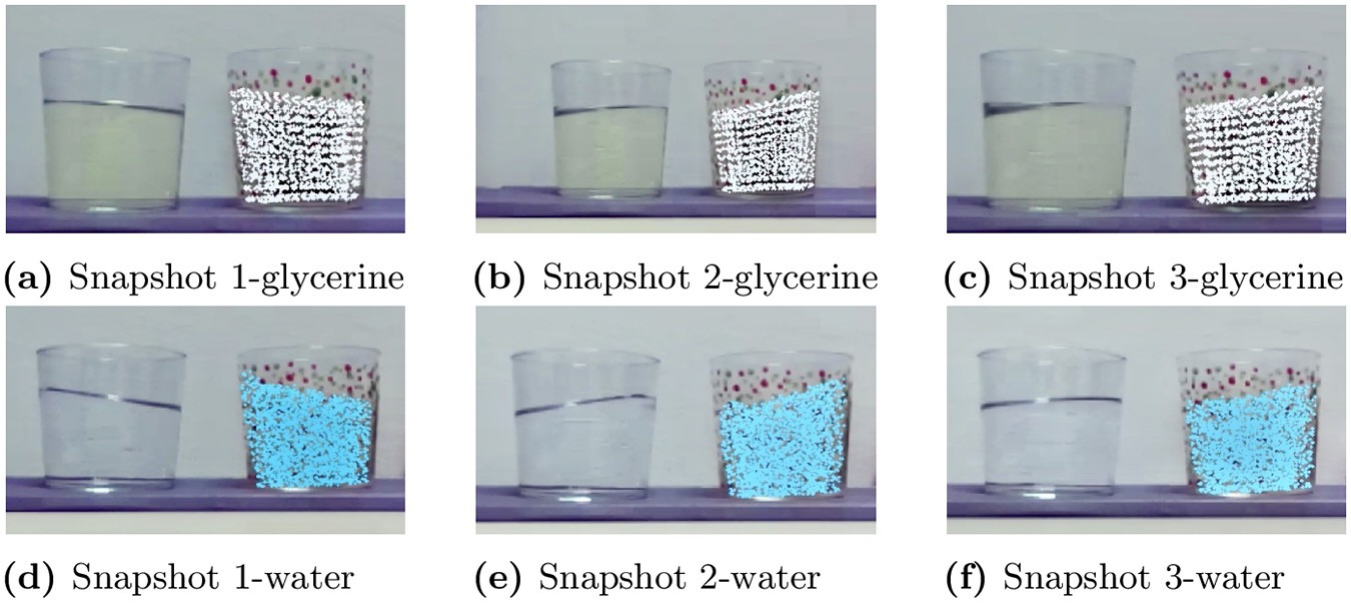
Snapshots employed for comparison between the real liquid and the digital twin. The free surface reconstruction has been evaluated to compute the error.

The resulting errors are shown in Table 3. Absolute value measurements are provided at *h*(0) and *h*(*l*) in mm for comparison. The error is also expressed in mm. They remain adequate according to the state-of-the-art in computer vision applications. The error grows with higher amplitudes of slosh, see Fig 8. Some sources of error could root in the approximation performed for velocity estimation. Remember that the pseudo experimental data with which we have built the model came also from a numerical approximation, SPH. Nevertheless, the resemblance is sufficient for learning a model, as well as corrections that will improve its performance.

**Table 3:** Numerical results of the experimental validation. Snapshot number refer to the ones shown in Fig 8.

WATER	*h*^*R*^(0)	*h*^*V*^(0)	*h*^*R*^(*l*)	*h*^*V*^(*l*)	mean error (mm)
Snapshot 1	5.52	11.32	5.38	8.28	0.7572
Snapshot 2	4.77	7.58	3.089	11.35	1.5254
Snapshot 3	3.694	2.839	2.839	6.53	1.0031
GLYCERINE	*h*^*R*^(0)	*h*^*V*^(0)	*h*^*R*^(*l*)	*h*^*V*^(*l*)	mean error (mm)
Snapshot 1	3.56	3.845	3.56	3.418	0.119
Snapshot 2	4.20	5.95	3.18	8.85	1.7428
Snapshot 3	3.11	3.53	2.54	7.77	1.249

## Conclussions and recommendations

In this paper we have presented and described a digital twin able to learn the sloshing dynamics occurring within a glass. We have shown that it satisfactorily reproduces the dynamics that a real stimulus would cause. The merge of online and realtime data acquisition, calculation and result representation has enabled realistic interaction between the two mediums.

The digital twin connects with the scene trough computer vision techniques based on feature extraction to obtain the velocity of the container. This is the input of the simulations that we have performed to obtain synthetic data with which the model has been built. Therefore, we establish a straight relationship between scene data and interpretable inputs for the twin.

Realtime performance has been achieved through the use of model order reduction techniques. *k*-PCA finds a space of 3-4 dimensions where the dynamics are embedded to perform the calculation with minimal loss of information. We have also proved the efficacy of GENERIC to learn more complex behaviors, such as viscoelasticity, widening the options that the twin offers.

While it has been difficult, in general, to obtain fully meaningful and quantitative comparisons with existing methods—that, in addition, focus on the pouring process, while we are interested in the sloshing phenomenon—, our method guarantees by construction the fulfillment of the laws of thermodynamics while bypassing the integration of Navier-Stokes equations. This has shown to provide very accurate results.

Undoubtedly, perception performance is a field of deep interest. New techniques constantly appear for achieving new learning challenges. Given these tools, and the results obtained from the classification training, new capabilities can be added to the twin. We expect to feed the algorithm with data of the free surface of a real fluid so as to distinguish the liquid properties of the liquid perceived. Also, the model could be corrected in case the liquid is unknown to go a step further and transform the model into a hybrid twin.

## Appendix—Stereo camera fundamentals

**Fig 9:**
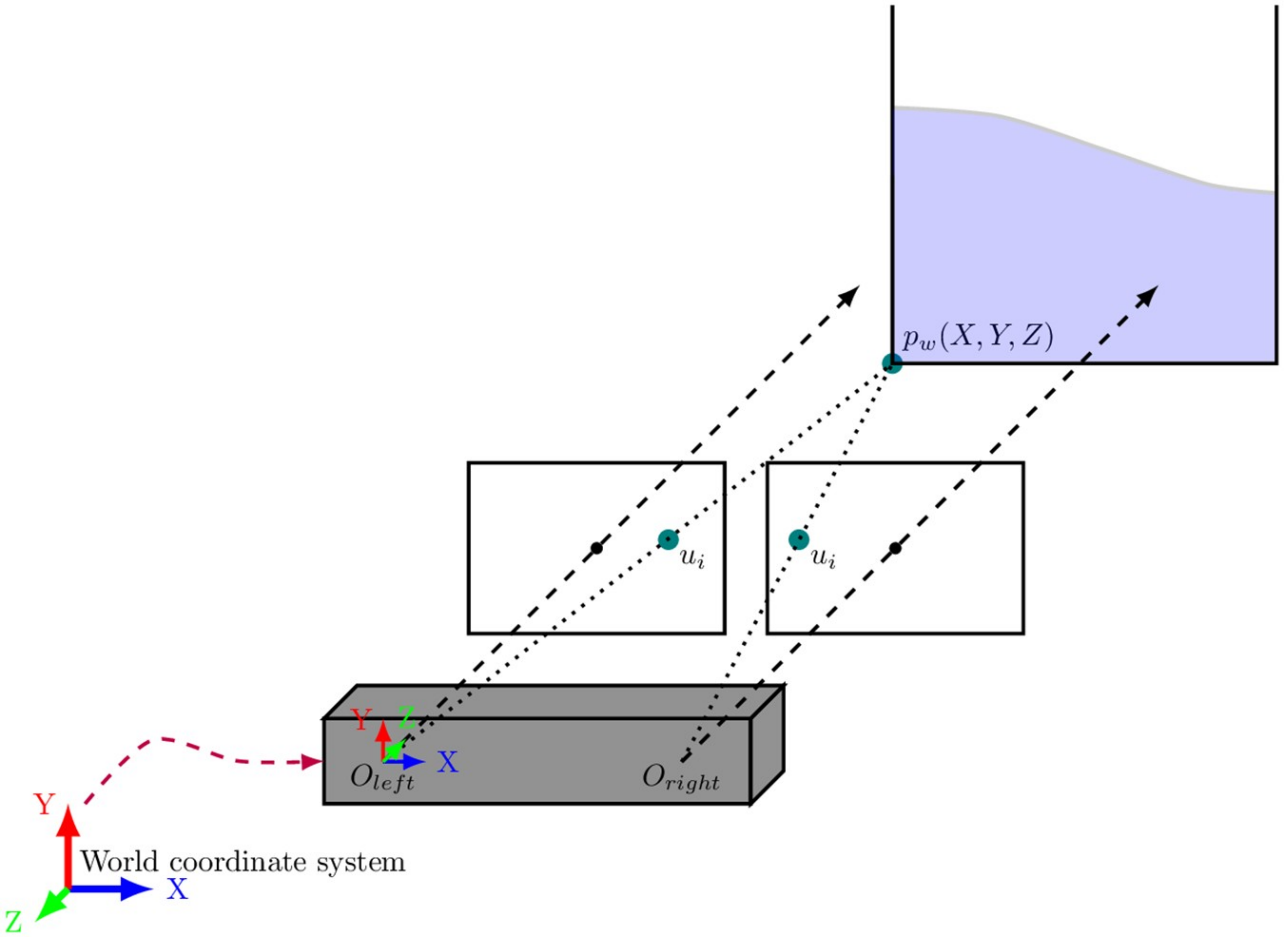
The picture shows the functioning of the stereo system. The camera moves freely, and its movement is related to the origin position through the extrinsic parameters. In computer vision, at least two images are required for the 3D reconstruction of a point. The camera performs continuous triangulations so as to export the depth of each pixel relating the 2D matches detected among right and left lenses.

The camera employed in our application is a Zed Mini model from Stereo Labs (https://www.stereolabs.com/zed-mini/). This camera incorporates a stereo system and an Inertial Measurement Unit, IMU. The camera is able to instantaneously provide the user with its intrinsic and extrinsic parameters, see Fig 9. This fact helps to speed up the computation of the inputs and outputs of our digital twin, as well as its augmented reality reconstruction.

The relationship between the 3D world and a 2D image, and viceversa, is build from the so-called intrinsic and extrinsic parameters. Intrinsic parameters relate the 2D position of a point, in pixel coordinates, with its 3D position with respect to the camera. Those parameters are the focal length of right and left cameras, and pixel spacing *s*_*x*_ and *s*_*y*_. They form the calibration matrix ***K***.

On the other hand, extrinsic parameters are those that represent the camera’s rotation (***R***) and translation (***t***) with regard to a reference coordinate system. With all this information, a point can be mapped from the real world to the pixel coordinates, and viceversa.

Having prior knowledge of the camera’s calibration and position, the 3D estimation of every point is performed through *triangulation* [48]. As mentioned before, the camera outputs the intrinsic and extrinsic parameters at each frame. As a result, we know the projection of the 2D features in the 3D real world system to perform any operation in the algorithm implemented.

$$\begin{array}{c} s[\begin{array}{c} u\\ v\\ 1 \end{array}]=[\begin{array}{ccc} f_{x} & 0 & c_{x}\\ 0 & f_{y} & c_{y}\\ 0 & 0 & 1 \end{array}][\begin{array}{cccc} r_{11} & r_{12} & r_{13} & t_{1}\\ r_{21} & r_{22} & r_{23} & t_{2}\\ r_{31} & r_{32} & r_{33} & t_{3} \end{array}][\begin{array}{c} X\\ Y\\ Z\\ 1 \end{array}] \end{array}$$

$$\begin{array}{c} {\tilde{x}}_{s}=K\left[R|t\right]p_{w} \end{array}$$

### Feature extraction

Transparent objects, such us those made of glass, have always entailed an extreme difficulty for feature extraction algorithms due to their lack of texture. Only recently techniques based on deep convolutional neural networks, CNN, have been developed to overcome this problem [49] [50].

In contrast, we aim to simplify the process for this application. Instead of using fiducial markers [22] or object alignment, we have added texture to the glass. Little points were painted in the glass to create points of interest that the feature detector could select. From the detection of these features, or points of interest, we localize the center of masses of the glass projected to its bottom surface.

We assume that we do not have prior knowledge of the position of the glass. We apply the Shi-Tomasi algorithm [51] for feature extraction in the area where the glass is expected to be. It finds the strongest, and more stable, features to track along the video sequence. The camera straightly provides the 3D position of the selected points of interest. With those points, we compute the center of masses of the glass projected to the bottom of the container. By tracking that point, we obtain information of the position and velocity of the glass.

## Supporting information

S1 Video Digital twin recordings. (TXT)
